# Rare Isolated Sphenoid Sinus Lesions: A Multi-case Study

**DOI:** 10.7759/cureus.73496

**Published:** 2024-11-11

**Authors:** Bassel Adra, Hamdoon AL Namani, Ajay Kumar Gona

**Affiliations:** 1 Otolaryngology-Head and Neck Surgery, Aintree University Hospital, Liverpool, GBR; 2 Otolaryngology-Head and Neck Surgery, Al Nahdha Hospital, Muscat, OMN; 3 Otolaryngology-Head and Neck Surgery, Bedfordshire Hospitals NHS Trust, Bedfordshire, GBR

**Keywords:** endoscopic sinus surgery, hamartoma, inverted papilloma, sinus lesion, sphenoid sinus, squamous cell carcinoma

## Abstract

Objective: To present clinical experiences in managing sphenoid sinus lesions at Al Nahdha Hospital, Oman with a focus on diagnostic challenges and surgical outcomes.

Methods: A retrospective analysis was conducted on six cases of sphenoid sinus lesions treated with endoscopic sinus surgery (ESS). Diagnostic modalities included contrast-enhanced computed tomography (CT) and magnetic resonance imaging (MRI), followed by histopathological confirmation.

Results: Six cases were included, of which four were diagnosed with inverted papilloma, with squamous cell carcinoma transformation in one of them, the fifth one was a case of sphenoid angiofibroma and the sixth one was sphenoid hamartoma. Malignant transformation into squamous cell carcinoma (SCC) was observed in one case. All patients underwent ESS without significant intraoperative or postoperative complications. Long-term surveillance is recommended.

Conclusion: Sphenoid sinus lesions are rare but require early intervention due to the potential for malignant transformation and proximity to critical neurovascular structures. High-resolution imaging, combined with endoscopic surgery, provides effective treatment. The recurrence risk for inverted papillomas, particularly with malignant transformation potential, necessitates prolonged follow-up.

## Introduction

Isolated sphenoid sinus lesions are rare, accounting for only 1-2.7% of paranasal sinus pathologies [1]. Lesions may present with nonspecific symptoms like headaches, vision abnormalities, postnasal drip, or nasal obstruction, often resulting in delayed diagnosis [2]. The deep anatomical location adjacent to vital structures such as the internal carotid artery, optic nerve, and cavernous sinus, makes early detection crucial [3]. Advances in high-definition endoscopic surgery and imaging techniques such as CT and MRI have improved the diagnosis and management of these conditions. This study shares our clinical experiences with a series of rare sphenoid sinus pathologies, with emphasis on diagnostic processes, surgical management, and outcomes.

## Materials and methods

Study design and inclusion criteria

This retrospective case series was conducted at Al Nahdha Hospital, a tertiary care center in Muscat, Oman, focusing on isolated sphenoid sinus lesions. Cases of isolated sphenoid sinus lesions confirmed via imaging and biopsy were included in the study. A cohort of six cases with sphenoid sinus lesions, including inverted papilloma, hamartoma, and angiofibroma were included in the study.

Preoperative imaging studies

Computed Tomography (CT) scans were used to assess bony erosion and anatomical relationships to adjacent neurovascular structures. Magnetic Resonance Imaging (MRI) was performed, and T1, T2, fluid-attenuated inversion recovery (FLAIR), and gadolinium-enhanced sequences were performed to evaluate soft tissue characteristics and lesion extent.

Histopathological analysis

Biopsy specimens from each lesion were subjected to histopathological examination to confirm the diagnosis and classify each lesion by cellular composition.

Surgical technique

Endoscopic sinus surgery (ESS) was performed for lesion resection, tailored to the specific characteristics of each case. Techniques employed included tumor resection, cauterization, coablation, and, in select cases, partial turbinectomy or septotomy to optimize surgical access. This study provides a detailed examination of each case, contributing to a deeper understanding of sphenoid sinus lesion management and patient outcomes.

## Results

Case 1: inverted papilloma of the sphenoid sinus

A 65-year-old man with an unremarkable medical history presented to the emergency department with a three-month history of intermittent left-sided nasal obstruction, recently complicated by episodes of vertigo and vomiting.

**Figure 1:**
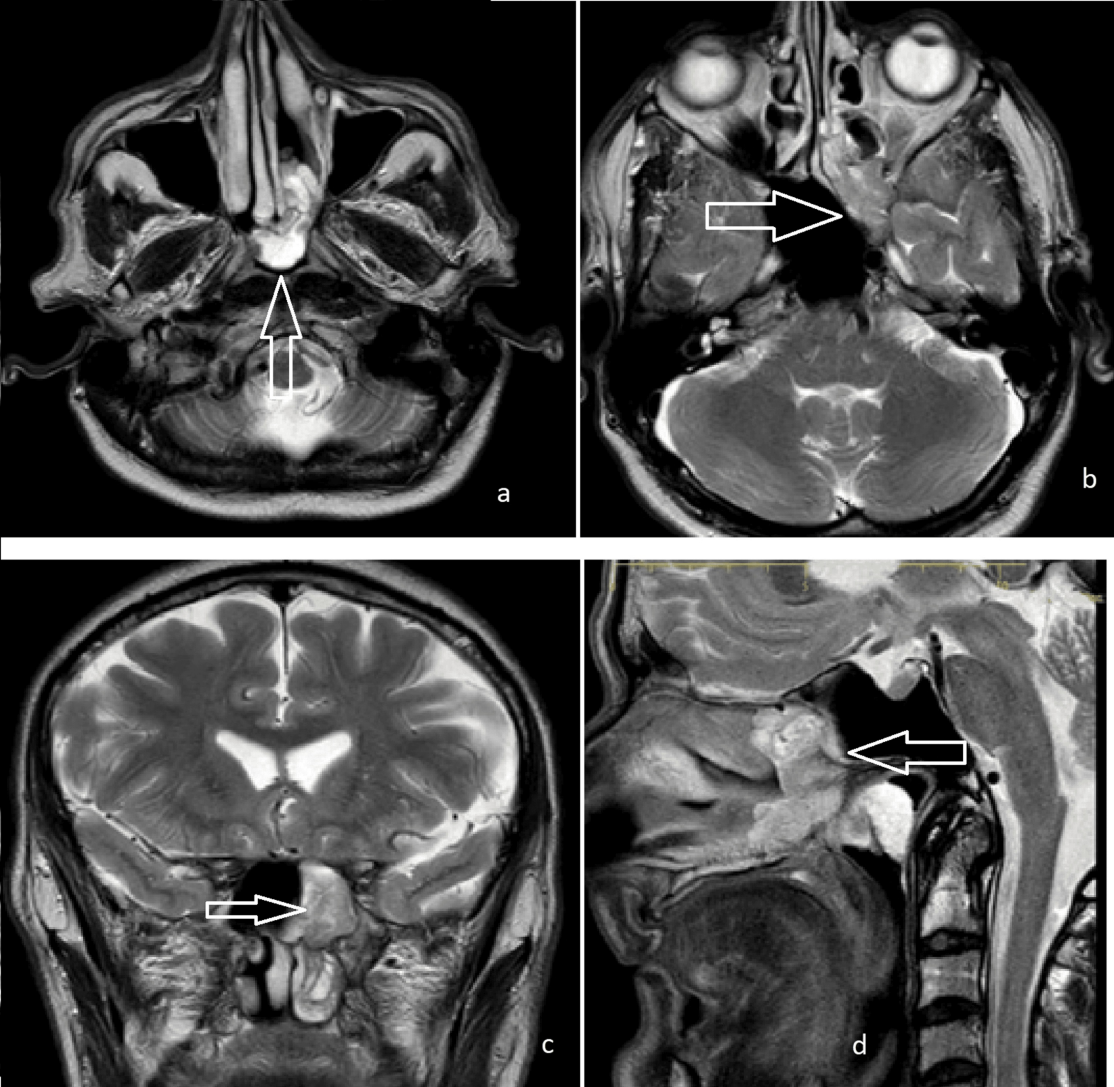
MRI scan images of the patient with isolated sphenoid (a,b) axial, (c) coronal, (d) sagittal views.

Imaging revealed a well-defined, enhancing soft tissue mass in the left sphenoid sinus measuring approximately 2.5 x 2.0 x 1.5 cm. The mass extended laterally through the sphenopalatine and ipsilateral pterygoid fossae, involving the ipsilateral nasal cavity. Bony erosion was evident near the left internal carotid artery, with optic foramen encroachment but no intraocular extension. Additionally, a polypoid, lobulated mass extended from the left nasal cavity through the posterior choana (Figure 1).

Case 2: inverted papilloma with squamous cell carcinoma transformation

**Figure 2:**
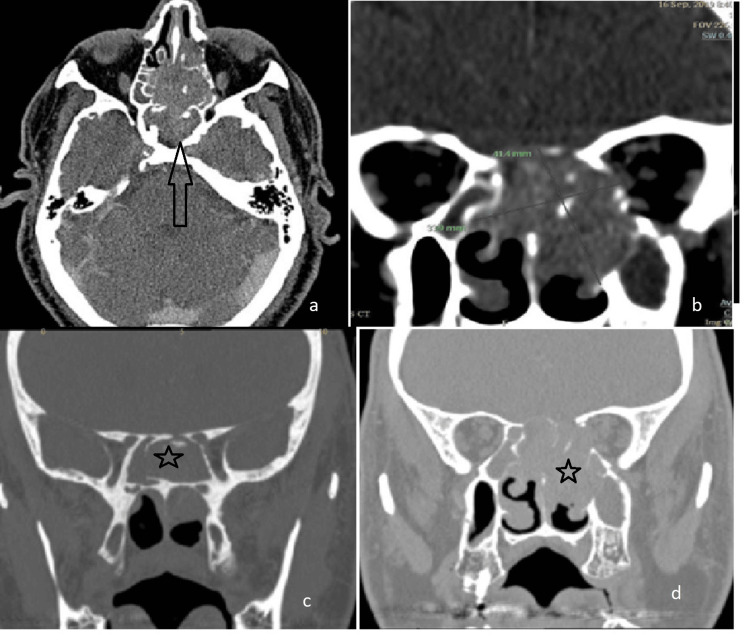
CT scan of sphenoid IP with SCC transformation (a) axial view, (b,c,d) coronal view. IP: inverted papilloma, SCC: squamous cell carcinoma.

A 64-year-old man with intact visual acuity and ocular motility presented to the outpatient clinic with a two-month history of left-sided nasal obstruction, intermittent epistaxis, and hyposmia, along with mild left-sided proptosis. Imaging revealed a large, aggressive mass in the left ethmoid sinus. The mass extensively involved the right nasal cavity, extraconal orbit, left maxillary sinus, left choana, and bilateral sphenoid sinuses, with predominant involvement on the left. Extensive bony destruction involved the left medial orbital wall, ethmoid sinuses, medial maxillary sinus wall, and cribriform plate. A bony defect was also identified in the anterior sella (Figure 2).

Initial biopsy findings indicated inverted papilloma with dysplasia. The patient underwent endoscopic sinus surgery for lesion resection. Intraoperatively, the mass was found to erode the left ethmoid air cells, middle and superior turbinates, and the inter-sinus septum, including the rostrum and posterior nasal septum. The rostrum exhibited significant erosion, necessitating partial resection. The mass was adherent to the sphenoid sinus roof, with the erosion of the sella and planum sphenoidale. Small areas of exposed dura were noted; however, there was no cerebrospinal fluid (CSF) leakage. Postoperatively, final histopathology confirmed transformation to squamous cell carcinoma. Consequently, the patient was referred to oncology and underwent adjuvant chemoradiotherapy.

Case 3: hamartoma of the sphenoid sinus

A 53-year-old male presented with a three-month history of right-sided headache, nasal obstruction, and nasal polyps without complaints of facial pain, anosmia, recurrent epistaxis, or other significant symptoms. Nasal endoscopy revealed a polypoid, fleshy mass originating from the sphenoid sinus and extending inferiorly to the middle turbinate in the posterior right nasal cavity.

**Figure 3:**
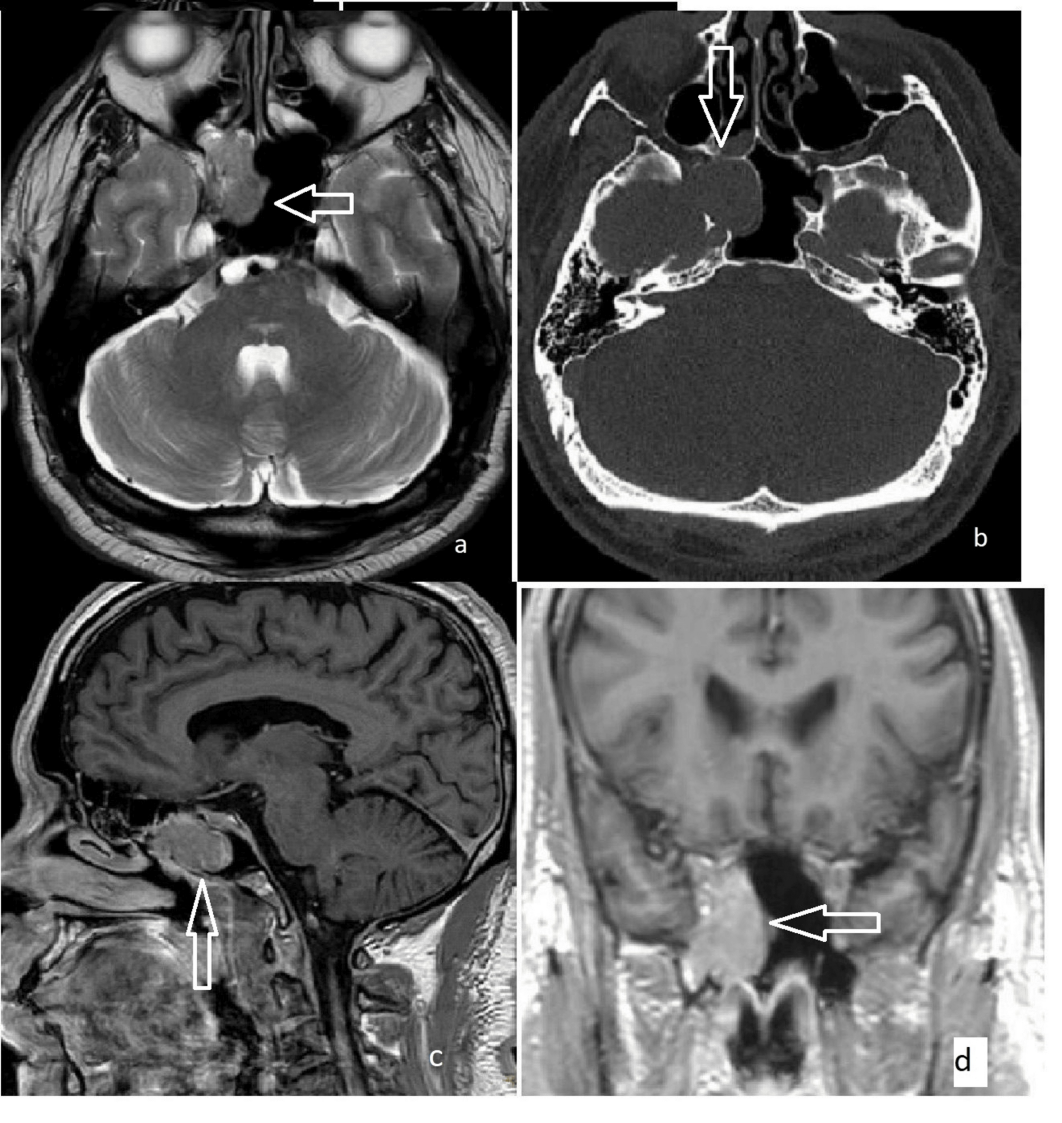
MRI images of the patient showing a hamartoma of the isolated sphenoid sinus (a) axial, (b) CT axial view, (c) sagittal, (d) coronal views.

Neuroimaging demonstrated a well-defined polypoid lesion within the right sphenoid sinus, measuring approximately 2.5 x 2.0 x 1.5 cm. The lesion exhibited heterogeneous T2 signal intensity, isointense T1, and FLAIR signal intensity, with mild heterogeneous enhancement post-gadolinium. Bony erosion was noted in the lateral wall of the sphenoid sinus, extending into the right temporal fossa, and in the inferior wall, extending toward the nasopharynx. No intracranial extension was observed (Figure 3).

The patient underwent functional endoscopic sinus surgery (FESS) for excision of the lesion. Intraoperatively, the lesion was found to originate from the floor of the right sphenoid sinus and the inter-sphenoid septum. Postoperative histopathological examination confirmed the diagnosis of sphenoid hamartoma.

Case 4: nasopharyngeal angiofibroma

A 37-year-old male presented with a one-year history of persistent right-sided nasal obstruction, intermittent epistaxis, hyposmia, mild headaches, recurrent thick nasal discharge, and occasional facial pain. Nasal endoscopy revealed a fleshy, polypoid mass filling the right nasal cavity with areas of necrosis and malodorous discharge. The mass extended across the midline, partially obstructing the choana and limiting visualization of the nasopharynx. Initial biopsy suggested lobular capillary hemangioma. For this reason, it was thought that the lesion did not need embolization or vascular imaging.

**Figure 4:**
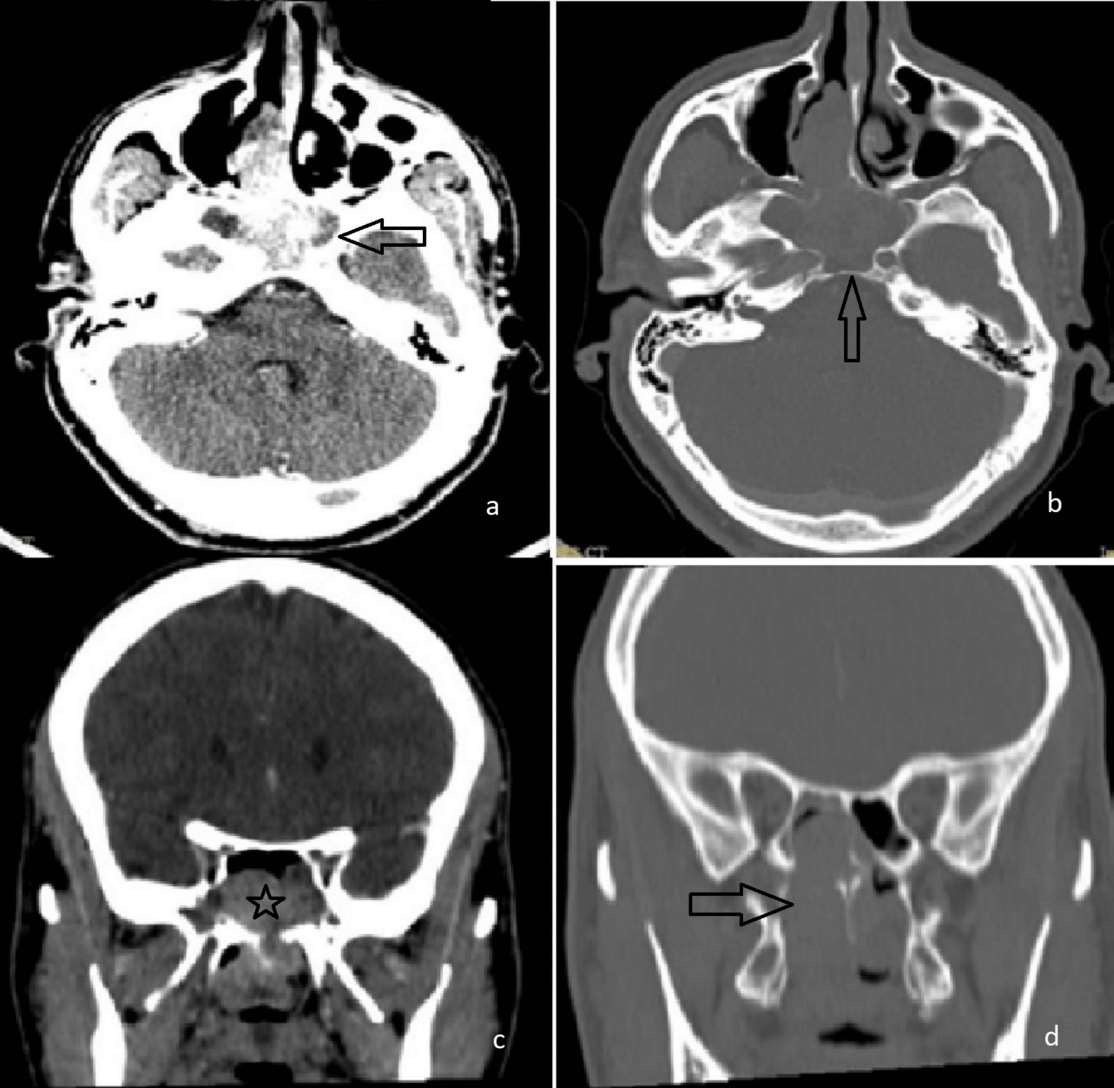
CT scan images of the sphenoid sinus-originated angiofibroma in the patient (a,b) axial views, (c,d) coronal views.

Neuroimaging demonstrated a heterogeneous, enhancing polypoid mass involving the right nasal cavity, nasopharynx, bilateral sphenoid sinuses, and, to a lesser extent, the right ethmoid air cells. Evidence of bony destruction was seen in the anterior and inferior sphenoid sinus walls, with thinning of the medial wall of the right maxillary sinus and the right hard palate. No intracranial extension was observed (Figure 4).

The patient underwent endoscopic sinus surgery with tumor resection aided by coablation. Intraoperatively, the moderately vascular polypoid tumor was found to originate from the floor of the anterior sphenoid sinus and the nasopharyngeal roof, extending into both sphenoid sinuses, the nasopharynx, and the posterior right nasal cavity. Debulking was achieved with coablation cautery and a debrider. For enhanced exposure, a posterior septotomy and partial middle turbinectomy were performed. Postoperative histopathology confirmed a diagnosis of nasopharyngeal angiofibroma.

Case 5: inverted papilloma of the sphenoid sinus in a young patient

A 23-year-old male presented with a two-year history of bilateral nasal obstruction, snoring, sleep apnea, and hyposmia, without complaints of nasal bleeding, headache, or visual disturbances. Nasal endoscopy revealed bilateral nasal polyps, with a larger polypoid lesion on the right side extending into the nasopharynx.

**Figure 5:**
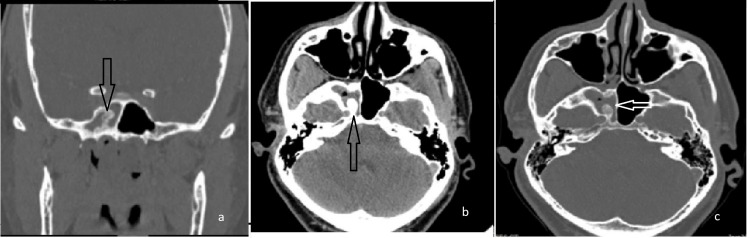
CT scan images of isolated sphenoid inverted papilloma in a young patient (a) coronal view, (b,c) axial views.

Imaging studies demonstrated a polypoid soft tissue mass within the right sphenoid sinus, measuring approximately 2.5 x 2.0 x 1.5 cm. The mass showed no contrast enhancement on computed tomography (CT) and no evidence of bony erosion or calcification (Figure 5).

The patient underwent functional endoscopic sinus surgery (FESS), including removal of the polypoid tissue and a partial middle turbinectomy. Intraoperatively, the lesion was found to originate from a bony projection in the right sphenoid sinus, which was partially resected. The mucosa was stripped and cauterized. Postoperative histopathological examination confirmed a diagnosis of inverted Schneiderian papilloma.

Case 6: recurrent inverted papilloma of the sphenoid sinus

A 53-year-old female presented with a 20-year history of frontal and occipital headaches. She had undergone two previous endoscopic sinus surgeries for inverted papilloma in India in 2009 and 2015. Her current symptoms included a two-month history of nasal obstruction, a one-year history of severe hyposmia, nasal clots, tinnitus, hearing loss, zygomatic and cheek numbness, neck pain, finger numbness, and occasional loss of consciousness.

**Figure 6:**
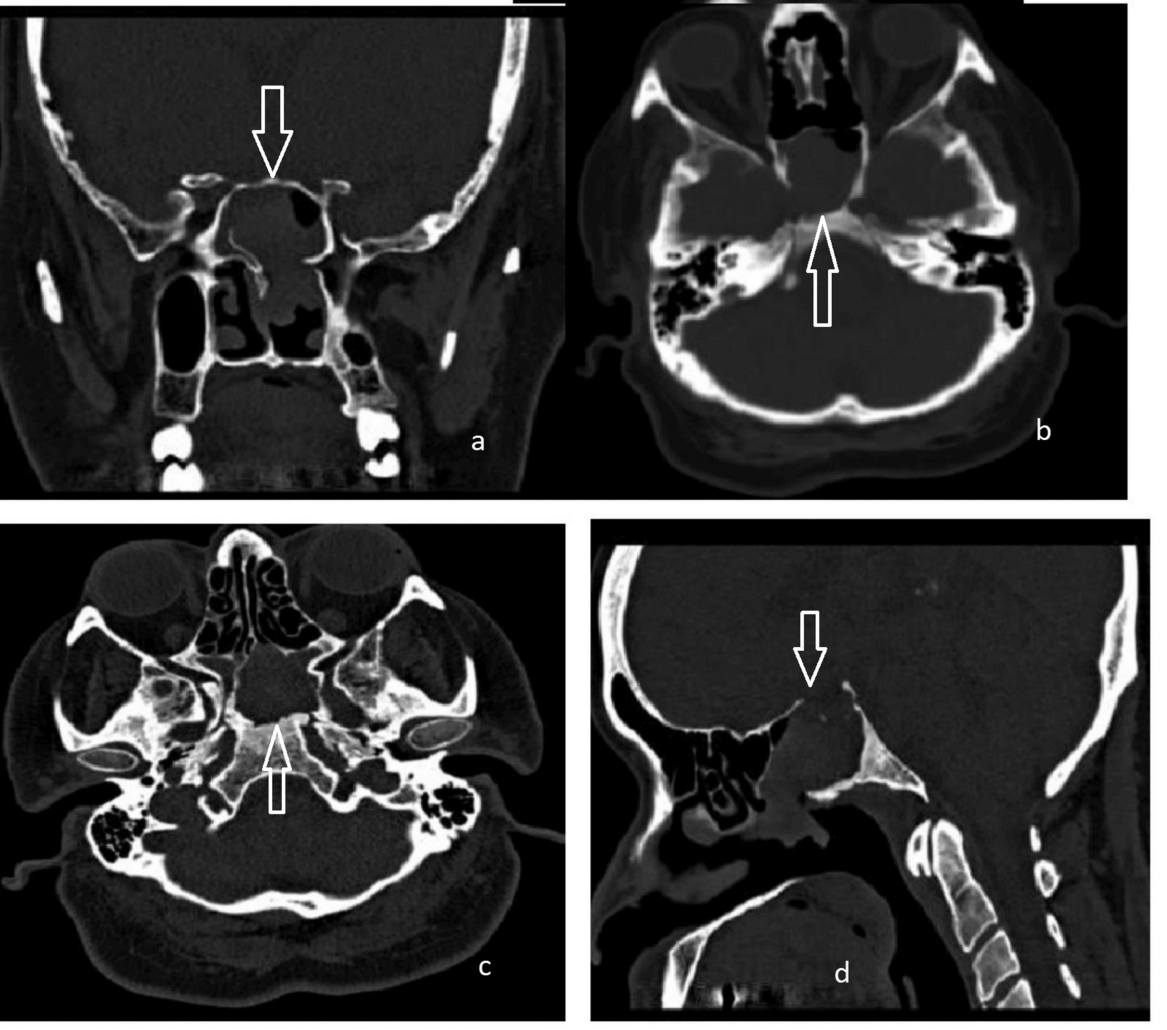
Third recurrent sphenoid inverted papilloma in a patient (a) coronal view, (b,c) axial view, (d) sagittal view.

Nasal endoscopic examination revealed a polypoidal mass in the left sphenoethmoidal region. Biopsy confirmed recurrent inverted papilloma. Imaging studies demonstrated a 2.5 x 2.0 x 1.5 cm mass within the sphenoid sinus, extending into the left ethmoid sinus and causing erosion of the sphenoid sinus walls. Minor erosion was also observed in the posterior clinoid process (Figure 6).

The patient underwent a third endoscopic sinus surgery for resection of the recurrent inverted papilloma. The surgical procedure involved endoscopic exposure of the sphenoid lesion, followed by the removal of the remnant anterior sphenoid wall, then identification of the polypoidal lesion originating from the posterolateral sphenoid sinus walls, with the identification of dehiscence in the bilateral parasellar carotids, right paraclival carotid, and posterior sella. After that, a resection of all diseased and polypoidal mucosa was performed until healthy bone was exposed, followed by cauterization of the affected area. Postoperative histopathological examination confirmed the diagnosis of recurrent inverted papilloma.

## Discussion

The deep anatomical location of the sphenoid sinus often delays early diagnosis of its pathologies, which may lead to serious complications due to proximity to vital structures [4]. Recent advancements in imaging and endoscopic techniques have significantly improved the diagnosis and management of sphenoid sinus lesions.

Isolated sphenoid sinus lesions generally present with headaches, followed by ophthalmological and nasal symptoms [5]. Of these lesions, 72% are inflammatory, 16% are neoplastic, and about 12% are related to other causes, such as cerebrospinal fluid leaks and fibrous dysplasia [6]. Although CT is the preferred initial investigation, distinguishing tumors from soft tissue swelling can be challenging, making MRI a useful complementary tool. We reviewed imaging findings from six cases with varying diagnoses, including a discussion of each pathological entity.

Inverted papillomas, as seen in the four cases presented in this study, are of particular concern due to their local aggressiveness, recurrence potential, and association with malignancy [7,8]. Recurrent disease and metachronous carcinoma can arise after long intervals [8]. Intracranial involvement is unusual and usually seen in recurrent cases [9]. In our cases, Endoscopic Sinus Surgery (ESS) was effective, though prolonged follow-up is critical due to the risk of recurrence and malignant transformation.

Sphenoidal sinus malignancies, although rare, require a multidisciplinary approach due to their potential for intracranial involvement. MRI and CT are essential in assessing these lesions' extent, differentiating tumors from retained secretions, and evaluating both bone erosion and adjacent soft tissue involvement [10-15]. The surgical approach should be tailored to each case, with careful consideration for neurovascular proximity and tumor origin.

Surgery remains the primary treatment for inverted papilloma, and careful intraoperative assessment and complete lesion resection are essential for successful outcomes. Close follow-up is necessary, as recurrences may occur months to years post-surgery [16]. The primary and preferred treatment of inverted papilloma is surgery. Advances in the surgical approach to the anterior skull base have resulted in increased opportunities for curing tumors involving these structures.

Sphenoid sinus hamartomas are uncommon, benign tumors that often present as asymptomatic or mildly symptomatic masses. They typically consist of respiratory epithelium or cartilage, as seen in respiratory epithelial adenomatoid hamartomas (REAH) and chondro-osseous respiratory epithelial adenomatoid hamartomas (COREAH). Symptoms may include nasal obstruction, sinusitis, or headache. Imaging studies usually reveal well-defined polypoidal lesions. Endoscopic resection is the standard treatment, with a low recurrence rate if complete removal is achieved. Differentiating these lesions from similar conditions like inverted papillomas is crucial to avoid unnecessary aggressive treatment [17,18].

Juvenile nasopharyngeal angiofibroma (JNA), while benign, is aggressive and often affects adolescents. In our adult case, ESS proved effective, though JNA’s vascular nature typically requires embolization in adolescent patients to minimize intraoperative bleeding risks [19,20].

However, this study has several limitations. First, the small sample size and retrospective design may limit the applicability of the findings to a wider population. Second, relying solely on imaging and tissue samples for diagnosis and follow-up might miss cases of recurrence that develop over a long period of time. Third, the lack of a control group prevents direct comparison between different treatment approaches. Finally, the inter-surgeon variability in surgical techniques may influence outcome consistency. To address these limitations, future research should use a larger sample size, a prospective design, and longer follow-up periods.

## Conclusions

Inverted papilloma confined to the sphenoid sinus is a rare condition, presenting diagnostic and therapeutic challenges due to its nonspecific symptoms and clinical presentation. Radiological assessments, particularly MRI and CT scans, are essential for accurately characterizing these tumors. Endoscopic sphenoidotomy remains the preferred treatment, offering effectiveness comparable to traditional external approaches but with fewer associated comorbidities.

Radiological and histopathological assessments, combined with meticulous surgical technique, contribute to effective management and reduce recurrence risk. Comprehensive, multidisciplinary management, especially for malignant cases, optimizes patient outcomes. Long-term follow-up is essential given the high recurrence risk for inverted papillomas, especially with potential malignant transformation.
